# Characterization of Oral Microbiota in Removable Dental Prosthesis Users: Influence of Arterial Hypertension

**DOI:** 10.1155/2017/3838640

**Published:** 2017-06-21

**Authors:** Leila Maria Marchi-Alves, Dayana Freitas, Denise de Andrade, Simone de Godoy, Adrielle Naiara Toneti, Isabel Amélia Costa Mendes

**Affiliations:** ^1^Ribeirão Preto College of Nursing, University of São Paulo, Ribeirão Preto, SP, Brazil; ^2^Clinical Hospital, Federal University of Triangulo Mineiro, Uberaba, MG, Brazil

## Abstract

**Introduction:**

Studies have described the possible relation between oral infections and atherosclerotic events.

**Objective:**

To characterize the oral microbiota of normotensive and hypertensive users of dental prostheses.

**Methods:**

The sample consisted of 41 complete dental prosthesis users, divided into groups: 21 participants with systemic arterial hypertension and 20 normotensive participants. The data collection included the characteristics of the sociodemographic variables and the determination of the microbial load in the saliva. For the descriptive analyses, Statistical Package for the Social Sciences was used. The description of the proportional differences between the groups was based on the application of Mann–Whitney's statistical test. Statistical significance was set at 5% (*p* < 0.05).

**Results:**

The analysis of the oral microbiota showed the vast growth of aerobic microorganisms in all samples from both groups. The microbial load of streptococci and staphylococci was significantly higher among hypertensive participants.* Candida* yeasts were detected in the saliva culture of most samples. The hypertensive participants rank in the category of very high colonization index/high risk of infection related to this microorganism.

**Conclusions:**

The mouth of dental prosthesis users, especially when hypertensive, can constitute an important reservoir of pathogens, indicating an established inflammatory or infectious condition or risk for developing this condition.

## 1. Introduction

The relation between oral and general health has been well documented in the literature. The study of the dynamic relation between the oral microbiota and the systemic inflammatory process is of particular interest [1]. Most studies have described the possible relation between chronic oral infections, mainly periodontal diseases, and thromboembolic and atherosclerotic events [1, 2]. Thus, the atherosclerotic vascular changes should be highlighted amidst the cardiovascular conditions associated with oral diseases [3, 4].

The correlation between the inflammatory process and the endothelial dysfunction with formation of atheromatous plaques can be important in the genesis of systemic arterial hypertension (SAH). The periodontal disease and SAH may also interact with the underlying inflammatory process that interferes in the endothelial function, aggravating the blood pressure (BP) control (PA) and favoring the development of lesions in target organs [5, 6]. Nevertheless, the mechanisms that link the cardiovascular and oral conditions have not been fully clarified yet.

Different microorganisms, such as bacterial strains and fungi, can interfere in the inflammatory process and in the formation of atherosclerotic plaque [7, 8]. The microbiota accompanying the absence of teeth also favors the formation of biofilms on the surface of oral cavity tissue [9]. The risk is especially exacerbated when the edentulous patients use a dental prosthesis, as the stay of artifacts in the oral cavity can significantly favor its microbial population [10].

The oral mucosa and the support tissue of the prosthesis undergo generally inflammatory changes, which can gain intensity across the period the device is used in. In addition, the structure and chemical composition of the material the dental prosthesis is made of favor the colonization by microorganisms. The prolonged contact of the colonized prostheses with the mucosa causes epithelial modifications that make the dental prosthesis user more susceptible to pathological events [11].

The events are aggravated by interactive mechanisms, such as unwashed, badly adapted, broken, or glued prostheses, which injure the patient's already sensitive mucosa and contribute to the proliferation of fungi and bacteria [12].

It should be highlighted that the presence of a prosthesis in the oral cavity leads to a decrease in the saliva flow and pH [13]. In addition, when the individual uses a complete dental prosthesis, the mucosa area covered by the prosthesis cannot have contact with saliva and impedes tissue cleaning by the mechanical action of the tongue, favoring the development of biofilms.

Overall, dental and dental prosthesis biofilm are similar in terms of complexity and microbial variation. The microflora found among dental prosthesis users is also multiple, with a prevalence of aerobic and anaerobic bacteria and yeasts [10].

Researchers have also observed that bacterial species frequently found in the periodontal area and strongly associated with systemic conditions can [9] be found in the mouth of edentulous prosthesis users or not [14].

Hence, we believe that the use of dental prostheses can affect the balance of the microbiological ecosystem in the mouth and lead to systemic changes. The objective in this study was to characterize the oral microbiota of normotensive and hypertensive users of removable complete dental prostheses.

## 2. Materials and Methods

Study developed at the Health Service of a Brazilian district, between July 2014 and March 2015. The study participants were the adult inhabitants of the place where the data were collected and users of removable complete dental prostheses. Nonprobabilistic sampling was used, including all prosthesis users living in the data collection site, who met the inclusion criteria and accepted to participate in the research. The target population consisted of 52 individuals.

The criteria adopted for exclusion were age younger than 18 years, having a natural dental element or any teeth replacement different from a removable complete dental prosthesis, smokers, alcohol users, recent use of antibiotics and corticosteroids, and diagnosis of autoimmune diseases, secondary hypertension, and diabetes mellitus.

Due to the exclusion criteria, the final sample consisted of 41 individuals using removable complete dental prostheses, divided into* Group 1*, 20 adults not diagnosed with SAH, and* Group 2*, 21 adults diagnosed with SAH.

The diagnosis of SAH was self-reported and confirmed by the registers in the patient history at the health service or the prescribed use of antihypertensive drugs.

Through an interview, the data on the sociodemographic variables were obtained. The oral microbiota was characterized through a culture of saliva samples for microbiological analysis and determination of the microbial load.

All saliva samples were collected early in the morning in a private room at the health service. The individuals were instructed to remain fasting for one hour and not use mouthwash for 12 hours before the collection. For the nonstimulated saliva collection, we used the spitting method [15]. We asked the participant to sit down with the head slightly bent downwards and try not to swallow or move the tongue and lips for one minute. Then, he expelled the saliva accumulated in the mouth (about 2.0 ml) inside test tubes.

The saliva samples were stored between 2°C and 8°C and transported in hard isothermal containers to the laboratory where the material was processed and analyzed.

The culture media used to analyze the microbiota in the saliva samples were Blood Agar (BA) and Tryptic Soy Agar (TSA) (BD® Difco™, Sparks, MD, USA), used to determine the microbial load of total aerobic microorganisms; Blood Agar enriched with menadione and hemin (ASK) and Tryptic Soy Agar (TSA) (BD® Difco™, Sparks, MD, USA), to determine the microbial load of total anaerobic microorganisms; Mannitol Salt Agar (MN) (BD® Difco™, Sparks, MD, USA) to isolate staphylococci; Mitis Salivarius Agar (MS) (BD® Difco™, Sparks, MD, USA), to determine the microbial load of oral streptococci; CHROMagar™ Candida (CR) (CHROMagar, Paris, FRA), to determine the microbial load of* Candida* spp.

For the microbiological processing, the saliva samples were submitted to two-minute agitation in a mixer (Mixtron-Toptronix/São Paulo, Brazil), at maximum speed for the purpose of homogenization. Next, they were submitted to decimal dilution series in saline solution up to 10^−4^. Volumes of 50.0 *μ*l of pure sample and of each dilution were seeded in Petri dishes with the media BA, ASK, MS, MN, and CR. The dishes with ASK were incubated in anaerobic condition, obtained by means of the Gaspak system (Probac, Brazil), in hermetically sealed jars (Permution, Curitiba, Brazil), for 7 to 10 days at 37°C. The BA dishes were incubated in aerobic conditions directly in an incubator at 37°C for 24 to 48 hours.

After plating and incubation for the appropriate time and temperature, the cells or small clusters grew in isolation, producing Colony Forming Units (CFU) of microorganisms. The CFU were counted in the appropriate dilution with the help of a stereoscopic microscope (Nikon®, Japan) and the quantities were registered to calculate the number of CFU per milliliter (ml) of sample.

The entire microbiological experiment was developed in a Class II Biological Safety Cabin – model BioSeg 12® (VECO Group - Campinas, Brazil).

The data were submitted to appropriate coding and typed through the elaboration of a code book in a Microsoft Excel worksheet in Windows XP (Microsoft Co, USA). For the descriptive analyses, the SPSS Statistical Software version 16.0 (SPSS Inc. Chicago, EUA) was used. The nonparametric Mann–Whitney test was applied to describe the proportional intergroup differences, comparing the variables of interest. In all analyses, statistical significance was set at 5% (*p* < 0.05).

The project received approval from the Ethics and Research Committee, guaranteeing compliance with National Health Council Resolution 466/12. The informed consent was obtained with subjects.

## 3. Results

Forty-one individuals participated in the study, 20 (48.8%) being normotensive and 21 (51.2%) hypertensive. The data on the sociodemographic description of the study population are presented in Table 1.

Among the normotensive participants, the mean age was 60.05 ± 7.51 years, against 65.57 ± 7.78 years among the hypertensive individuals (*p* = 0.026). All individuals included in the non-White category stated they were mulatto or black.

In the normotensive group, the mean Systolic Blood Pressure (SBP) and Diastolic Blood Pressure (DBP) corresponded to 127.85 ± 14.59 mmHg and 81.5 ± 10.16 mmHg, respectively. Among the hypertensive participants, the SBP equaled 159.52 ± 19.91 mmHg and the DBP 86.04 ± 9.24 mmHg.

The bacterial growth results in selective culture media for anaerobic microorganisms, streptococci, staphylococci, and* Candida* spp. are expressed in Table 2.

No microorganism growth was observed in the selective culture media for anaerobic microorganisms (ASK culture medium), in both groups. Streptococci and staphylococci were detected in all samples analyzed (100%). Except for the absence of anaerobic microorganisms, the lowest percentage growth of microorganisms was found in the selective culture medium for* Candida *spp., present in 71.4% of the samples from hypertensive participants and in 60% of the saliva samples from normotensive individuals.

In Table 3, the data on the microscopic counts of CFU/ml are displayed, representing the microbial load of total aerobic microorganisms (BA culture medium), streptococci (MS culture medium), staphylococci (MN culture medium), and* Candida* spp. (CR culture medium).

When comparing the results of the saliva sample cultures obtained in the hypertensive group with the samples collected from the normotensive individuals, a statistically significant difference was observed in the microbial load of streptococci (*p* < 0.01), cultivated in the medium MS and staphylococci (*p* < 0.05), cultivated in the medium MN.

## 4. Discussion

The predominance of the female gender indicates that, in the study population, women use removable complete dental prostheses more frequently than men. Experts have already indicated a positive association between the female gender and edentulism [16]. It should be highlighted, however, that our research did not intend to assess the need to use prostheses, but the actual use of the device. It has been argued that women have greater access to medical and dental services, are more open and receptive to health-related themes, and are more attentive to the factors that may negatively affect their physical integrity [17, 18].

In addition, the higher frequency of women in this research can result from the feminization of old age [19], as most participants in our study are elderly. The increased age is also positively associated with edentulism [20, 21], a condition that indicates the need for a complete prosthesis.

We observe, however, that the predominant age range in the hypertensive group is superior to that in the normotensive group (*p* = 0.026). The prevalence of younger individuals in the normotensive group can be justified by the higher incidence of pathological events directly associated with age progression [22].

Concerning the marital family situation or living condition, according to some authors, married life, associated with other psychosocial factors, is directly linked to the rise or drop in pressure levels [23, 24].

As for the skin color, the sample was predominantly categorized as White or Caucasian. Most studies, differently, evidence that non-White individuals, mainly Afro-Americans, are at greater risk of developing arterial hypertension [25, 26]. Other authors found similar results, with a majority of White individuals in the hypertensive group, but argued that this fact can be rebuttable, as White individuals commonly have greater control over their health or clinical conditions [27].

Considering the prevailing education level in both groups, we believe that low education levels are associated with high ratios of tooth loss or edentulism, as indicated by other authors [28, 29]. What the profession/occupation variable is concerned, most participants in both groups indicated doing housework, being traditionally qualified as “housewives.” This finding can be justified by the prevalence of elderly women in the sample characterization.

Concerning the analysis of the oral microbiota, in all samples in both groups we observed the vast growth of aerobic microorganisms. The microbial load of streptococci and staphylococci was significantly higher among the hypertensive participants.

*Candida* yeasts were detected in the saliva culture of most samples from the normotensive and hypertensive participants, particularly the* albicans *strain, which was the most frequent in both groups. The hypertensive patients rank in the category of very high colonization index/high risk of infection related to this microorganism.* Candida albicans* was the most frequent species in the global sample.

The growth of streptococci and staphylococci was significantly higher in the saliva samples of hypertensive patients when compared to the microbial load of the same microorganism identified in the samples from the normotensive group. Considering the maxima and medians, the extent of the figures is impressive, especially concerning the uncountable CFU of streptococci.

Considering the median in the study groups and the parameters described in the literature [30–32], we identified that, for the microorganism* Candida,* the hypertensive participants rank in the category of very high colonization index/high risk of infection.

Hence, our results indicate that the mouth of edentulous dental prosthesis users, especially when hypertensive, can constitute a reservoir of pathogens as important as the oral cavity of teethed individuals, keeping in mind that individuals in normal health conditions can present up to 10^10^ of bacteria in the oral cavity [33].

It has been discussed that the role of oral cavity bacteria in the etiopathogenesis of other conditions can be due to the migration of the bacteria to the extraoral infection focus or to the establishment of a chronic systemic inflammatory condition based on the infection located in the mouth [34]. In case of hypertension, the situation can worsen, as the permanent rise in pressure levels can lead to systemic changes that are closely related to oral problems, such as reduced saliva flow, reduced protein concentration in the saliva, increased quantity of neutrophils, increased parathyroid hormone levels, abnormal vitamin D metabolism, and reduced calcium concentration and absorption [35].

All events mentioned favor the oral inflammatory process, which in turn can aggravate the hypertension through mechanisms such as platelet activation, increase in inflammatory biomarkers, and endothelial dysfunction [36]. Hence, it should be investigated whether the changes in the composition of the oral microbiota are causal factors of hypertension, resulting from the disease, or whether both situations are true.

The oral changes associated with advanced age are further aggravating factors of this condition, such as soft tissue injury and flaccidity, dry mouth, and coated tongue. The oral tissue loses elasticity, flake, and become more fragile and susceptible to oral cavity injuries, favoring the colonization by opportunistic microbiota [37]. It should also be kept in mind that the cognitive, functional, or neuropsychiatric problems that can affect the elderly make it even harder to perform the oral hygiene [38]. It should be reminded that the hypertensive participants in the sample are on average almost six years older than the normotensive participants and, therefore, more predisposed to such events.

In another study to assess the predominant colonization on the dental prostheses of the elderly, 18 bacterial species were identified in the biofilms of the prostheses assessed, the most frequent being* Streptococcus* spp.,* Candida* spp., and* Neisseria* spp. [39]. Similarly to our findings, Baena-Monroy et al. [40] concluded that the microorganisms* Candida albicans*,* Staphylococcus aureus,* and* Streptococcus mutans* frequently colonize the oral mucosa of dental prosthesis users, but also identified that this condition is more frequent among individuals diagnosed with prosthetic stomatitis.

We know that* Streptococcus* species are considered compatible with the host as they are primary colonizers of the oral cavity and do not present a highly toxic or virulent profile, being therefore more related to health than to disease conditions [34]. Under adverse conditions, however, they may become pathogenic.

*Candida* yeast is another saprophytic microorganism that, depending on predisposing factors, becomes pathogenic and can provoke local and superficial inflammatory manifestations or even systemic infections that can lead to death [41]. In our study, positive cultures for* Candida* were present in 71.4% of the saliva samples from the hypertensive participants and in 60% of the samples from the normotensive participants.

The dental prosthesis has served as a predisposing element for the oral colonization by bacteria and fungi, due to the relation between the oral hygiene habits, prosthesis cleaning, the material the device is made of, and the behavior of the microorganism [42, 43]. In addition, the natural affinity of the* Candida *strains to colonize the acrylic material the prosthesis is made of is exacerbated by the presence of bacteria through the coaggregation phenomenon [44, 45]. On the other hand, the bacterial microbiota can produce antifungal substances and regulate fungal growth by competing with the yeasts to use the nutrients [46].

The isolated or associated presence of these events favors the disequilibrium between the microorganism and host, compromising the latter's defense and permitting the disordered growth of the fungus and the tissue invasion, characteristics of the opportunistic infectious disease [47].

Our study did not intend to perform a previous evaluation of the participants oral health condition. We do not know the previous history of diseases and lesions in the oral cavity and the conditions or habits of hygiene of the prosthesis and the mouth, which may characterize a limitation of this work.

## 5. Conclusions

Based on our results, the mouth of edentulous dental prosthesis users, especially hypertensive individuals, can constitute an important reservoir of pathogens, indicating an established inflammatory or infectious condition or risk for developing this condition. It is a matter for further investigation whether the changed composition of the oral microbiota is a causal factor of hypertension, results from the disease, or both.

Thus, we consider that oral health recovery and promotion actions need to include specific groups in a differentiated manner, such as chronic patients and dental prosthesis users. To develop these actions, we believe that the multiprofessional and interdisciplinary approach offers greater probability of success. Intersectoral actions to strengthen individuals and communities in setting priorities and plan and implement strategies to achieve health are also fundamental.

## Figures and Tables

**Table 1:** Numerical and percentage distribution of normotensive (NT) and hypertensive (HT) participants according to sociodemographic variables. Brazil, 2015.

Variables	NT (*n* = 20)		HT (*n* = 21)		Total (41)	
	*n*	%	*n*	%	*n*	%
*Gender*						
Female	18	90	18	85.7	36	87.8
Male	02	10	03	14.3	05	12.2
*Age range (in years)*						
45–55	05	25	01	4.8	06	14.6
55–65	08	40	06	28.6	14	34.2
65–75	07	35	12	57.1	19	46.3
75–	—	—	02	9.5	02	4.9
*Marital family situation*						
With partner and children	07	35	08	38.1	15	36.6
With relatives without partner	07	35	07	33.3	14	34.1
With partner without children	02	10	05	23.8	07	17.1
Living alone	04	20	01	4.8	05	12.2
*Skin color*						
White	15	75	17	81	32	78
Non-White	05	25	04	19	09	22
*Education (in years of study)*						
0 (no education)	05	25	06	28.6	11	26.8
1–5	13	65	10	47.6	23	56.1
5–10	02	10	05	23.8	07	17.1
*Profession/occupation*						
Housewife	09	45	13	61.9	22	53.7
Retired	06	30	07	33.3	13	31.7
Others	05	25	01	4.8	06	14.6

**Table 2:** Growth of anaerobic microorganisms, streptococci, staphylococci, and *Candida *spp. in saliva samples of normotensive and hypertensive users of removable complete dental prostheses. Brazil, 2015.

Groups	Microorganisms	Growth microorganisms	*n*	%
Normotensive	Anaerobic	Positive	—	—
		Negative	20	100
	Streptococci	Positive	20	100
		Negative	—	—
	Staphylococci	Positive	20	100
		Negative	—	—
	*Candida*	Positive	12	60
		Negative	08	40

Hypertensive	Anaerobic	Positive	—	—
		Negative	21	100
	Streptococci	Positive	21	100
		Negative	—	—
	Staphylococci	Positive	21	100
		Negative	—	—
	*Candida*	Positive	15	71.4
		Negative	06	28.6

**Table 3:** Minimum, maximum, and median values of Colony Forming Units (CFU) of microorganisms in different saliva culture media of normotensive and hypertensive users of removable complete dental prostheses. Brazil, 2015.

Group	Culture medium	Minimum (CFU/ml)	Maximum (CFU/ml)	Median (CFU/ml)	*p* ^*∗*^
Normotensive (*n* = 20)	BA	8	8.16*E* + 7	3.27*E* + 6	
	MS	28	5.92*E* + 7	970	
	MN	<0.001	1.92	0.005	
	CR	<0.001	9.4*E* + 5	2.00	

Hypertensive (*n* = 21)	BA	60	1.98*E* + 8	2.84*E* + 7	0.134
	MS	460	1.08*E* + 10	1.16*E* + 7	0.009
	MN	<0.001	1.60*E* + 8	0.040	0.025
	CR	<0.001	1.64*E* + 6	800	0.236

^*∗*^Mann–Whitney test. Selective culture media: BA: Blood Agar; MS: Mitis Salivarius Agar; MN: Mannitol Salt Agar; CR: CHROMagar.
